# Advances in Cardiovascular Wearable Devices

**DOI:** 10.3390/bios14110525

**Published:** 2024-10-30

**Authors:** Sheikh Muhammad Asher Iqbal, Mary Ann Leavitt, Imadeldin Mahgoub, Waseem Asghar

**Affiliations:** 1 Department of Electrical Engineering & Computer Science, Florida Atlantic University, Boca Raton, FL 33431, USA; siqbal2019@fau.edu (S.M.A.I.); mahgoubi@fau.edu (I.M.); 2 Asghar-Lab, Micro and Nanotechnology in Medicine, College of Engineering and Computer Science, Boca Raton, FL 33431, USA; 3 Christine E. Lynn College of Nursing, Florida Atlantic University, Boca Raton, FL 33431, USA; mleavit3@health.fau.edu; 4 Department of Biological Sciences (Courtesy Appointment), Florida Atlantic University, Boca Raton, FL 33431, USA

**Keywords:** cardiovascular, wearable devices, telehealth monitoring, implantable devices

## Abstract

Cardiovascular diseases are a leading cause of death worldwide. They mainly include coronary artery disease, rheumatic heart disease, andcerebrovascular disease, and. Cardiovascular diseases can be better managed and diagnosed using wearable devices. Wearable devices, in comparison to traditional cardiovascular diagnostic tools, are not only inexpensive but also have the potential to provide continuous real-time monitoring. This paper reviews some of the recent advances in cardiovascular wearable devices. It discusses traditional implantable devices for cardiovascular diseases as well as wearable devices. The different types of wearable devices are categorized based on different technologies, namely using galvanic contact, photoplethysmography (PPG), and radio frequency (RF) waves. It also highlights the use of artificial intelligence (AI) in cardiovascular disease diagnostics as well as future perspectives on cardiovascular devices.

## 1. Introduction

Cardiovascular diseases, commonly known as heart diseases, are a group of diseases related to the heart and blood vessels [1]. Cardiovascular diseases include coronary artery disease, rheumatic heart disease, and cerebrovascular disease, and [1]. They are the leading cause of death in the world. Each year, around 17.9 million people die from cardiovascular disease in the world. Approximately 695,000 deaths were due to heart disease in the US in 2021, which is about one in every five deaths [1,2]. One reason for the increase in cardiovascular disease mortality is the limited availability and accessibility of adequate diagnostic and monitoring tools. Traditional diagnostic tools are not only expensive but are also not sufficient to address the rapid progression of cardiovascular disease. A major risk factor for cardiovascular disease is an unhealthy lifestyle, which includes a lack of physical activity, an unhealthy diet, and the use of alcohol and tobacco [1]. According to the Centers for Disease Control and Prevention (CDC), from 2018 to 2019, USD 239.9 billion has been spent on the treatment of cardiovascular disease [2]. Cardiovascular disease can be better managed with the continuous and real-time monitoring of the vital parameters related to cardiovascular disease. These parameters include but are not limited to heart rate, electrocardiogram (ECG), oxygen saturation in the blood (SPO2), activity status, and blood pressure (BP). Wearable devices are now widely being used for this purpose [3,4,5,6,7].

Wearable devices can be worn on the skin or on clothing and they offer the low-cost, real-time, and continuous monitoring of the target biomarkers [3]. Most of the wearable devices consist of a receptor and a transducer. A receptor detects the target, and the transducer converts the detection into a usable signal [3]. Moreover, wearable devices are in accordance with the World Health Organization (WHO) ASSURED (Affordable, Sensitive, Specific, User-friendly, Rapid, robust, Equipment-free, and Deliverable to end-users) criteria to offer monitoring and diagnostic tools at the point of care (POC) settings [8]. According to one estimate, there were around 1.1 billion connected wearable devices around the world in the year 2022 [9]. Most of the wearable devices are for fitness and wellness purposes that include monitoring parameters related to cardiovascular disease [10]. These wearable devices use different technologies for monitoring different diseases. These technologies include photoplethysmography (PPG), radio frequency (RF) waves, and galvanic contact with the skin-based sensors.

In this article, we review wearable devices for cardiovascular disease. For this purpose, we will review some existing traditional devices and discuss some of the wearable devices and their integrated technologies for cardiovascular disease. The article will also highlight future directions for wearable devices, specifically in cardiovascular disease.

## 2. Implantable Cardiovascular Devices

**Figure 1:**
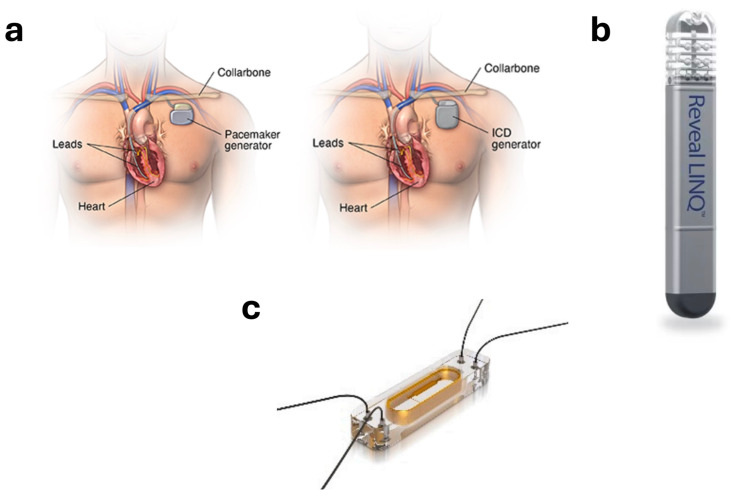
(**a**) Pacemaker and ICD [32]. (**b**) Medtronic Reveal LINQ insertable cardiac monitor [30]. (**c**) CardioMEMS for monitoring pulmonary artery pressure [23].

Traditionally, implantable devices have widely been used for monitoring patients with cardiovascular disease. Some of the most frequently used implants are pacemakers and implantable cardioverter defibrillators (ICDs). Recently, implants like CardioMEMS™ and insertable cardiac monitors have also been introduced for diagnostic purposes. Pacemakers, as shown in Figure 1a, are used for restoring the heartbeat in the case of arrhythmias [11]. Arrhythmia is a condition in which the heart beats with an irregular rhythm, either faster (tachycardia) or slower (bradycardia) than normal [11,12]. A pacemaker continuously monitors the heart’s rate and rhythm and when needed, sends an electrical signal to normalize the heartbeat. It is implanted on the left side of the chest near the collarbone. Similarly, an ICD is an implant slightly larger in size than a pacemaker (due to a larger battery) but has more features. An ICD also continuously monitors the heart’s rate and rhythm but differs in that it can deliver an internal shock to stop a lethal arrhythmia [13]. Over the years, implantable devices have seen advancements in their size and technology. One such technology is insertable cardiac monitors (ICMs), also known as implantable loop recorders (ILRs) that insert cardiovascular devices with minimal incision. An implantable loop recorder is a small device to record the electrocardiogram (ECG) and it is used to detect and diagnose arrhythmias that are not apparent on examination [14,15,16,17,18]. A cardiac loop recorder, is one such ILR [19]. Unlike pacemakers or ICDs, it does not restore the heart rhythm; it is only used to record the ECG for an extended period for diagnostic purposes. It can record the ECG for as long as three years [14]. It helps providers to diagnose tachycardia or bradycardia so the correct device or treatment can be determined. Medtronic Reveal LINQ (USA), as shown in Figure 1b, is another ILR. It is one of the world’s smallest ILRs at 44.8 mm × 7.2 mm × 4.0 mm size and has a battery life of 3 years [20]. One of the arrhythmias it can detect is atrial fibrillation (AFib). AFib can lead to blood clots and increase the risk of stroke, heart failure, and other cardiovascular conditions [21]. CardioMEMS™ (Abbott, USA), as shown in Figure 1c, is another implantable solution for monitoring heart failure (HF) using pulmonary artery pressure. In HF, the heart is unable to pump sufficient blood to fulfill the needs of the body [4]. Pulmonary artery pressure increases as HF worsens and CardioMEMS™ monitors these changes in the pulmonary artery pressure so that providers can intervene before patients need to go to the hospital [22,23]. CardioMEMS is implanted permanently in the distal pulmonary artery with a safe right heart catheterization procedure. A pressure sensor inside CardioMEMS then measures changes in the pulmonary artery pressure, a reflection of the retention of fluid in the lungs due to worsening heart failure [24].

Implantable cardiovascular devices are very useful in monitoring cardiovascular disease; however, they have limitations. The devices mentioned above are costly and are not accessible to everyone. A pacemaker costs between USD 20,000 to USD 100,000 depending on the type of the pacemaker [25]. Similarly, a simple ICD costs USD 18,000 in the US [26]. A cardiac loop recorder costs around USD 11,329 and a Medtronic Reveal LINQ costs around USD 2542 [27,28]. A CardioMEMS™ average cost is around USD 17,750 [29]. Moreover, not everyone is eligible for these implants; for example, an ICD is only recommended for HF patients with reduced ejection fraction, meaning their heart pump is weak and they are at risk for lethal arrhythmias. Similarly, CardioMEMS is indicated for NYHA Class II or III heart failure patients who either have been hospitalized for heart failure in the previous year and/or have elevated natriuretic peptides [24]. Patients with preserved ejection fraction are not eligible for an ICD [4]. Not only are these implants costly but they also involve the risk of surgery. All these implants are inserted with a major or minor incision. Most of them have a limited lifespan; for example, Reveal LINQ is only suitable for three years [30]. Similarly, pacemakers and ICDs have lithium batteries and require an implantable device replacement procedure around every five to ten years for replacing the battery [31].

Due to power limitations and the burden of additional surgery for battery replacement, most of these implants do not always report parameters continuously. They rather report the daily averages of the parameters. Wearable devices, on the other hand, being non-invasive in nature, can be used to monitor vital parameters related to cardiovascular diseases in real-time and continuously; however, most wearables are not for diagnostic purposes like ICDs or lCMs. The subsequent paragraphs discuss some of the existing and emerging wearable devices for different cardiovascular conditions. The next section also highlights different technologies that are most used in cardiovascular wearable devices for measuring cardiovascular parameters.

## 3. Cardiovascular Wearable Technologies

Cardiovascular wearable devices have seen advancements over the years. Different technologies have been used in these wearables. The most frequently used technologies are photoplethysmography (PPG), radio frequency (RF), and wearables using galvanic contact. This section discusses these technologies and discusses some of the most commonly used as well as emerging cardiovascular wearable devices.

### 3.1. Galvanic Contact

Galvanic contact is one of the oldest methods for measuring cardiovascular parameters. In this method, galvanic contact is made with the skin. Galvanic contact can be used to measure cardiovascular parameters such as ECG and HR by detecting potential differences generated by the heart. Cardiopulmonary parameters such as thoracic impedance, respiration rate, and lung fluid can be measured using galvanic contact by measuring the change in the applied signal due to the changes in these parameters in the thoracic region. The electrical signal can either be a voltage or current signal.

### 3.2. Photoplethysmography (PPG)

Photoplethysmography is widely used for detecting cardiovascular parameters. It is based on the absorption and scattering of optical light by the blood in peripheral circulation [33]. It consists of a light-emitting diode (LED) and an optical detector. The optical detector detects changes in the intensity of the light emitted by the LED. Changes in light intensity are affected by the volumetric changes in the blood flow. These volumetric changes can then be used to detect different cardiovascular parameters including heart rate (HR), heart rate variability (HRV), blood pressure (BP), stroke volume, and oxygen saturation in the blood (SPO_2_).

### 3.3. Radio Frequency (RF) Waves

Radio frequency (RF) waves are electromagnetic waves in the frequency spectrum of 3 Khz to 300 MHz. RF waves get distorted when they are obstructed by human organs. Their obstruction can be used to detect different cardiovascular parameters including HR, respiration rate, blood pressure (BP), and lung fluid. The next section will describe the use of these technologies in different wearable devices for measuring different cardiovascular parameters.

## 4. Cardiovascular Wearable Devices

Cardiovascular wearable devices have been used to detect different bio-signals related to cardiovascular disease. These wearable devices can be categorized into two major categories: body-mounted wearables and smart flexible wearables. Body-mounted sensors are mounted on different parts of the body to measure the target bio-signal. They have the sensor packaged inside the mounted casing which is then attached to the body. On the other hand, smart flexible wearables have sensors on the flexible substrate, with conductive traces, which are directly attached to the body. The next section discusses the applications of both types of CWDs for measuring significant cardiovascular parameters including ECG, BP, thoracic impedance, and HR.

### 4.1. Electrocardiogram

Electrocardiogram (ECG) is a vital and one of the most common cardiovascular parameters for monitoring and diagnosing different cardiovascular diseases. ECG is the electrical representation of the heart with a PQRS complex, reflecting the flow of electrical signals through the heart. The ECG is usually measured using galvanic contact with the skin using electrode leads. Traditionally, a 12-electrode system is used for measuring ECG. A traditional ECG monitor is not wearable and hence is not portable. Both body-mounted CWDs and flexible CWDs are used for measuring ECG.

**Figure 2:**
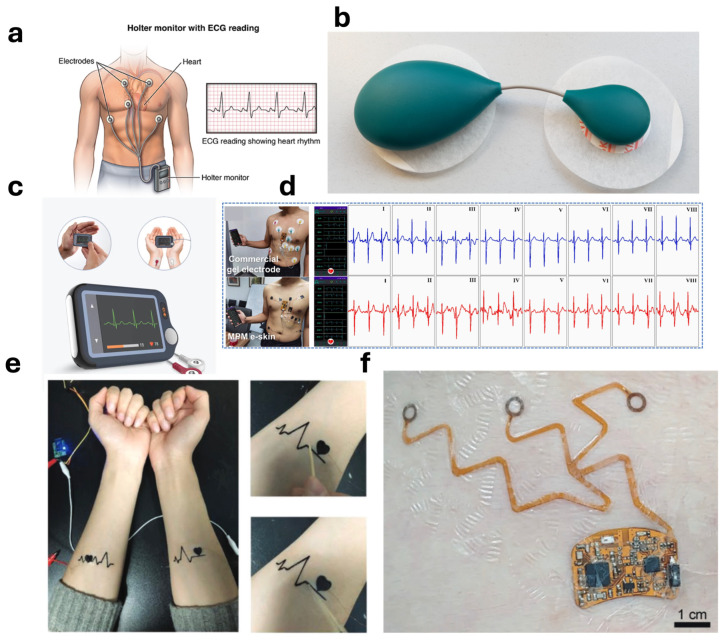
(**a**) A Holter monitor with multiple electrodes [34]. (**b**) An ECG monitor by SAVVY [35]. (**c**) A palm-sized ECG monitor by Wellue [37]. (**d**) A 12-lead ECG wearable on e-skin along with its comparison [38]. (**e**) A multifunctional, self-healable e-skin wearable for measuring ECG signal [39] (**f**) Flexible ECG monitor with non-stretchable components [40].

A Holter monitor is a common example of a body-mounted wearable for measuring ECG. A Holter monitor, as shown in Figure 2a, is a portable, external, multi-electrode CWD for measuring ECG over 24 h or longer [34]. SAVVY (Ljubljana, Slovenia) is another body-mounted ECG monitor, as shown in Figure 2b, that measures ECG in real-time and continuously [35]. It has a battery life of measuring ECG for 500 weeks and can be charged in 2 h [36]. It is connected to a mobile application called MobECG that allows the real-time visualization of the ECG waveform and can be sent to the medical practitioner [36]. The monitor is suitable for the detection of AFib, and can help in preventing cryptogenic stroke [36]. In patients with atrial flutter and atrial fibrillation, blood can stagnate and clot in the left atrium; if the clot dislodges, it can travel to the brain and cause a stroke.

A personal ECG monitor by Wellue, as shown in Figure 2c, is a two-electrode system for measuring ECG. It is a palm-sized monitor that can measure ECG for 24 h continuously [37]. The smart watch made by Apple also allows the measurements of the ECG. It is a three-electrode system that allows the measurement of the ECG from the wrist where the user is required to press the button on the dial using the other hand. The watch is capable of measuring ECG comparable to a single-lead ECG [38]. In a study conducted, ECG from the Apple Watch was found to be 98.3% sensitive towards the classification of AFib and 99.6% specific with respect to the normal sinus rhythm classification [39].

Cui et al. have developed an intelligent 12-lead electrocardiogram monitor with 8 channels that can monitor ECG for 12 h daily [41]. The ECG monitor, as shown in Figure 2d, is an electronic skin (e-skin) made with MXene/Polyurethane mesh (MPM). E-skins are epidermal-based wearable devices that involve the direct attachment of electronics to the skin like a tattoo. The wearable is trained using long short-term memory and convolutional neural network to diagnose four arrhythmias with over 99% diagnostic accuracy [41]. Similarly, Wang et al. have developed a multifunctional, self-healable e-skin wearable for measuring different vital signs including ECG. This wearable, as shown in Figure 2e, is made up of a combination of graphene/silk fibroin/Ca^2+^ (Gr/SF/Ca^2+^) [42]. The wearable is robust to deformations and adheres to the skin tightly. It was able to detect the PQRST complex of the ECG, as shown in Figure 2e [42]. Kim et al. have also reported a stretchable and breathable e-skin wearable for monitoring the ECG [40]. The wearable, as shown in Figure 2f, is based on stretchable and breathable medical adhesives with non-stretchable components integrated into it. The sensor has been reported to comfortably monitor ECG for five days and the signal was also transmitted wirelessly to a smartphone using a low-energy Bluetooth connection [40]. The wearable was also programmed with an R-peak detection algorithm where the R-peaks correlate with the R-peaks of the measured ECG signal. The R-peaks were then used to measure respiration rate and heart rate [40].

### 4.2. Heart Rate

Heart rate is an important cardiovascular indicator and can be indicative of different cardiovascular conditions. It is dependent on the contraction of the heart and hence reflects the rate at which the heart is pumping blood. A healthy individual should have a heart rate in the range of 60–100 beats per minute (BPMs); however, in different cardiovascular conditions, it can be lower or higher than the normal range. For example, in heart failure and hypertension, the heart rate may be higher than usual. There are two ways to measure heart rate: electrical and PPG. In electrical measurements, usually, heart rate is measured using an electrocardiogram or ECG. R-R peak detection and Pan/Tompkins algorithms are suitable algorithms to measure heart rate using ECG [43].

**Figure 3:**
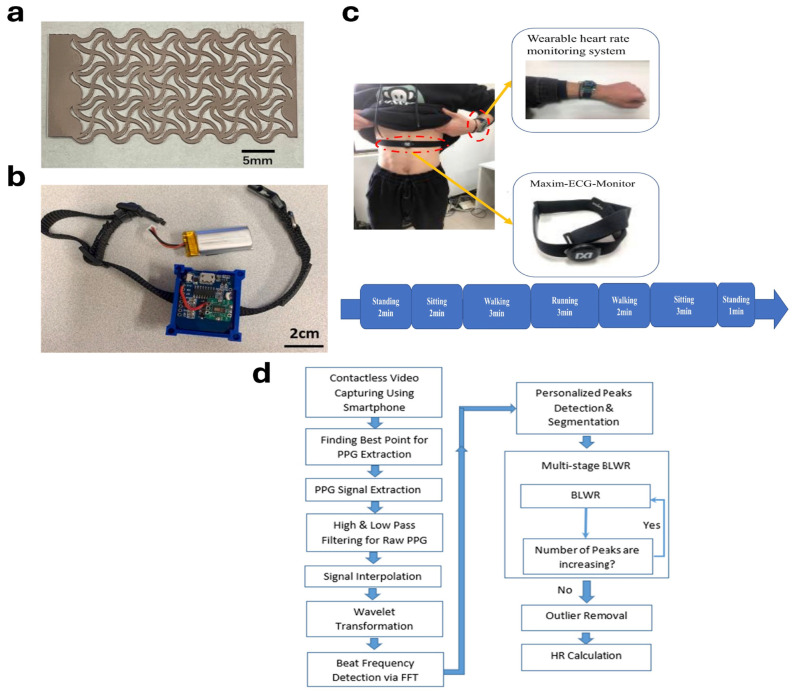
(**a**) A piezoelectric-based e-skin heart monitor [44]. (**b**) A PPG-based heart rate monitor for equine [47]. (**c**) The framework of an accelerometer-based removal of motion artifact from the PPG signal for heart rate measurement [48]. (**d**) The flowchart diagram of remote PPG (rPPG) using camera [51].

Modern electrical-based HR wearables use piezoelectric sensors to convert the heartbeat into electrical signals for measuring HR. One such wearable has been developed by Ji et al. [44]. They have developed a heart rate monitor using a piezoelectric film with a serpentine layout [44]. The wearable, as shown in Figure 3a, is highly stretchable and sensitive e-skin. It is only 168 μm in thickness with a voltage sensitivity of 0.97 mV/με [44]. It is attached to the chest where the strain sensor captures the chest vibrations and converts them into electrical signals for measuring heart rate and respiration rate [44]. Similarly, Mokhtari et al. have also developed a heart rate wearable based on piezoelectric theory [45]. The wearable is a portable cardiac monitor that monitors heart rate and pulse pressure. The wearable is based on a piezoelectric sensor that converts the sounds of the heartbeat into HR. It is a lightweight system of 50 g with a thickness of 2 cm, length of 5 cm, and width of 3 cm [45]. The wearable was correlated with the standard heart rate monitor and both values matched within an error of 3% [45]. Most of the commercially available HR wearable devices, for example, Oura Ring, Apple smartwatch, and Fitbit, are based on PPG technology.

Monitoring the heart rate with PPG uses lights with longer wavelengths for deeper penetration [46]. For this reason, red and green lights are most frequently used. Red light has deeper penetration into the tissues; however, it is more prone to motion artifacts; therefore, green light is also preferred in the PPG-based HR wearables [46]. Miller et al. have developed a non-invasive heart rate monitor for horses [47]. Heart rate measurements at rest from the wearable, as shown in Figure 3b, were compared with those measured by a stethoscope and were found to be 94% correlated [47]. A peak detection algorithm was used to measure heart rate from the PPG signal for this purpose [47]. PPG signals are prone to motion artifacts; therefore, they are usually cleaned using accelerometers [48]. Gao et al. have proposed one such technique where HR measurements are cleaned using the signal from an accelerometer [49]. Gao et al. have developed a wearable device that combines the signal from an accelerometer with the PPG signal to remove noise from it using a least-squares algorithm. The overall framework of the device is shown in Figure 3c [49]. PPG signals are also distorted with muscle artifacts and are, therefore, cleaned using an electromyogram (EMG) signal. Friman et al. have developed one such wearable. The wearable uses both an accelerometer signal along with an EMG signal to increase the accuracy of HR. The study has found that the EMG signal inclusion for the removal of muscle artifacts reduces HR estimation error by 49% in comparison to the use of an accelerometer alone [50]. New techniques include the use of remote PPG (rPPG) for the measurement of HR. Hosni et al. have used a camera to measure PPG remotely [51]. The PPG signal is processed using different signal processing techniques including high and low pass filters followed by Mexican heat wavelet transformation [51]. In this study, the PPG was calculated using the change in the green color of the pixel in two consecutive video frames. The videos of the face were recorded at 0.4 m and for 30 s [51]. The flowchart of the experiment is shown in Figure 3d. The study shows that HR can be measured using rPPG with a mean absolute error of 3.58 and a standard deviation of 2.4 [51].

### 4.3. Blood Pressure

Blood pressure is an important parameter in cardiovascular disease, as it indicates the force with which blood is flowing through blood vessels [52]. A blood pressure reading usually measures both systolic pressure, the pressure in the arteries during the contraction of the heart, and diastolic pressure, the pressure when the heart is at rest or in the relaxation phase [52]. A healthy blood pressure is less than 120 mmHg systolic and 80 mmHg diastolic [52]. On the other hand, hypertension measures higher than this range (systolic pressure 140 mmHg or higher and diastolic pressure 90 mmHg or higher). Hypertension is a leading cardiovascular disease with approximately 1.28 billion adults 30–79 years old affected with hypertension worldwide [53].

The first non-invasive BP apparatus, a sphygmomanometer, was developed by von Basch in 1881, and in 1896 Scipione Riva-Rocci developed a sphygmomanometer based on an inflatable arm band connected to a mercury column, similar to what is being used today with the cuff-based BP measurement [54,55]. Initially, the sphygmomanometer was only able to measure the systolic blood pressure but since then BP technology has seen many advancements. Traditional BP apparatuses are cuff-based and therefore are not very comfortable because the cuff is inflated until the artery is obstructed. Moreover, they are not portable enough to be carried or used regularly. For this reason, cuffless wearable BP apparatuses have been developed to conveniently measure BP continuously [56]. Most of these wearable BP monitors use different techniques such as PPG, bioimpedance, and capacitive pressure values to measure BP readings.

**Figure 4:**
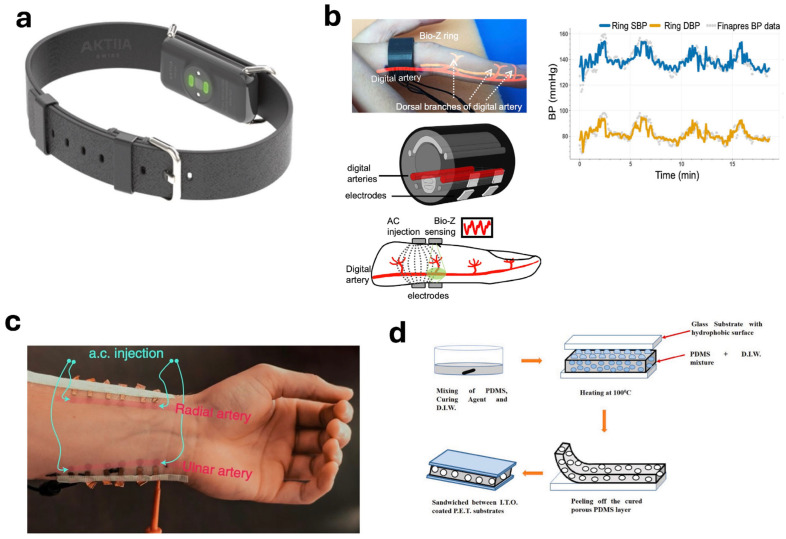
(**a**) A cuffless BP monitoring bracelet, Aktiia, that measures BP using PPG signal [57]. (**b**) A bioZ ring to measure BP non-invasively using bioimpedance along with the BP values from the ring comparison with a standard Finapres BP values [58]. (**c**) A flexible e-skin-based BP monitor based on graphene electronic tattoo [60]. (**d**) A capacitive transducer e-skin pressure sensor [61].

Aktiia (UK) is one such cuffless non-invasive BP monitor that measures BP using the PPG technique [57]. Aktiia, as shown in Figure 4a, is a bracelet that houses optical sensors to measure the BP from the wrist. It is 20 g in weight and can measure BP for over a week on a single charge [57]. The bracelet uses an algorithm subject-specific value of a subject to calibrate the device [56]. This calibration is only valid for 30 days and requires recalibration every month [56]. Almeida et al. have conducted a study to compare the accuracy of the Aktiia with the traditional ambulatory BP apparatus [56]. The study has found no significant differences between the two monitors, as daytime BP measurements from Aktiia were found to be comparable to those of a standard ambulatory BP monitor [56]. Similarly, Sel et al. have also developed a cuffless BP ring [58]. The ring, as shown in Figure 4b, consists of electrodes and measures BP using the bioimpedance technique. The ring is not sensitive to skin tones, unlike most of the optical sensors. BP was estimated from the changes in bioimpedance signal based on the volumetric changes due to pulsatile blood flow [58]. The ring estimates BP with a low error (mean ± standard deviation) of 0.11 ± 3.87 mmHg for diastolic and 0.11 ± 5.27 mmHg for systolic [58]. The BP values obtained from the ring were found to correlate with the BP values from a medical-grade finger cuff BP monitor with high Pearson’s correlation coefficients of 0.76 for SBP and 0.81 for DBP, as shown in Figure 4b [58]. These figures are in accordance with the standards set by International Standard Organization 81060-2 that require that the mean value of the differences in the determinations shall be within or equal to ±5 mmHg (±0.67 kPa), with a standard deviation not greater than 8 mmHg (1.07 kPa) [59].

Similarly, Kireev et al. have used bioimpedance to measure BP values. For this purpose, they have developed a graphene-based flexible BP monitor [60]. Flexible BP monitors are an advancement in cuffless BP monitors [60]. They are printed on flexible substrates to form e-skin. The GET, as shown in Figure 4c, is a lightweight self-adhesive and measures BP continuously using electrical bioimpedance [60]. Three sets of GET pairs were placed on the wrist over the radial and ulnar arteries to measure bioimpedance (Z). While measuring Z, the control BP values of the participant were also measured using a medical-grade BP monitoring device Finapres NOVA. The ΔZ values reflect the volumetric changes in the artery due to pulse pressure waves. The ΔZ is then correlated to the BP values as arterial volume is inversely proportional to ΔZ and a generally higher BP results in higher arterial volume [60]. The GET was able to measure BP for more than 300 min and measured systolic pressure with an error of 0.2 ± 5.8 mmHg and diastolic pressure with an error of 0.2 ± 4.5 mmHg. These results are comparable to the grade A classification for BP monitoring [61]. Similarly, Bijender et al. have also proposed a potential flexible BP monitor made up of polydimethylsiloxane (PDMS) [61]. The sensor, as shown in Figure 4d, is a capacitive pressure sensor that uses a capacitive transduction mechanism with flexible polyethylene terephthalate (PET) electrodes with PDMS encapsulated between them. The PET electrodes are coated with indium-tin-oxide (ITO) and have an operating pressure range of 0 mmHg (1 Pa) to 750 mmHg (100 kPa). It has been found suitable for measuring BP by detecting the oscillometric wave generated by the capacitive pressure sensor when pressure is applied in the BP range of 55–220 mmHg [61]. This experiment was measured by applying pressure in the BP range of 55–220 mmHg using a BP machine and not by actual volumetric changes due to blood flow.

As discussed, wearable BP monitors use different parameters such as bioimpedance or capacitive pressure readings to measure the pressure changes in the artery. Such wearable BP monitors often require calibration with a standard BP monitor to map these readings from the sensors with the BP readings. Moreover, BP readings are also subject-specific and therefore require calibration with the BP readings of a subject to calculate BP from the parameters measured by the wearable BP monitor.

### 4.4. Thoracic Fluid Index

**Figure 5:**
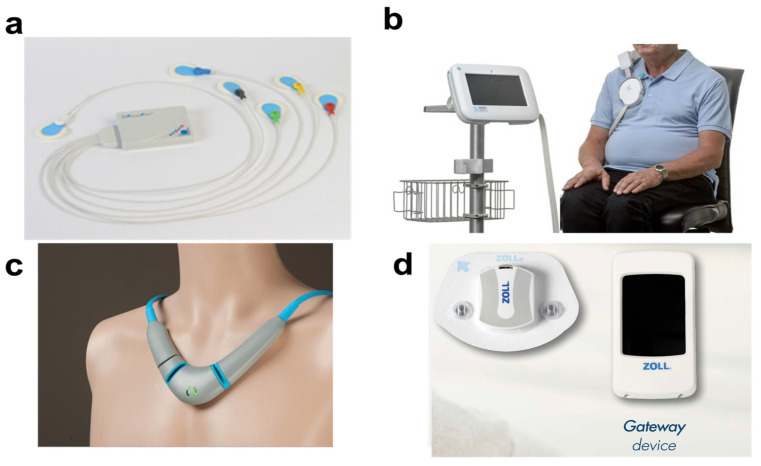
(**a**) PhysioFlow for measuring impedance cardiography [64]. (**b**) Remote Dielectric Sensing Vest for monitoring thoracic fluid index using electromagnetic beam [66]. (**c**) CoVa 2 for measuring thoracic fluid index and other hemodynamic parameters [67]. (**d**) Microcore for measuring thoracic fluid index and other cardiovascular parameters including ECG, HR, activity, and posture [68].

Thoracic fluid index is a measurement of the intrathoracic fluid [62]. It is an important estimation in monitoring heart failure. Heart failure is one of the leading cardiovascular diseases affecting approximately 64 million patients worldwide [2]. It is difficult to treat HF; however, it can be better managed by guideline-directed medical therapy and the vigilant monitoring of the patient’s condition [63]. Traditionally, thoracic fluid index can be measured using implantable devices like an ICD or CardioMEMS™; however, different body-mounted wearable sensors have been developed in the recent past to measure thoracic fluid index. PhysioFlow(France) is one such body-mounted non-invasive device [64]. PhysioFlow, as shown in Figure 5a, consists of six thoracic surface electrodes and measures different hemodynamic parameters such as stroke volume, cardiac output, and impedance cardiography [64]. These are the parameters that help in thoracic fluid management. Similarly, Remote Dielectric Sensing (ReDS) by Sensible Medical(NC, USA), as shown in Figure 5b, is another wearable that measures lung fluid content using electromagnetic beams [65,66]. ReDS has been found to reduce re-hospitalization rates by 48% in a group of 268 patients [66]. Both PhysioFlow and ReDS are not very convenient to be worn; therefore, much more compact and compatible wearable devices have been developed for thoracic fluid index monitoring.

CoVa 2 (MI, USA) is one such device that measures thoracic fluid index, cardiac output, stroke volume, and ECG [66]. CoVa 2, as shown in Figure 5c, is a necklace-shaped wearable that measures hemodynamic parameters using chest bioimpedance using two electrodes [66]. The values from CoVa 2 are communicated through using the Patient Gateway device. It has been found to predict heart failure events based on the thoracic fluid index and cardiac stroke volume. According to the study, the thoracic fluid index increases by more than 40% before all the HF events and stroke volume decreases by 8% in 60% of acute decompensated heart failure cases [66,67]. Similarly, Microcore (µCor) by Zoll (Chelmsford, MA, USA) is another body-mounted wearable that measures thoracic fluid index [68]. The µCor, as shown in Figure 5d, uses radiofrequency to measure the thoracic fluid index. The measurement of the thoracic fluid index is based on the changes in the interstitial edema where changes in interstitial edema are based on the strength and changes in signal path delay [69]. It also measures other important cardiovascular parameters including respiration rate, activity, HR, ECG, and posture [68]. These parameters are sent to a medical care team using WiFi using a Patient Gateway device that receives data from the sensor using Bluetooth [68,70]. According to the study conducted on 522 patients, patients with thoracic fluid index being monitored by their clinicians using µCor were found to be 38% less likely to be hospitalized than the patients whose thoracic fluid index was not monitored by their clinicians [68].

## 5. Artificial Intelligence for Cardiovascular Diseases

Most of the cardiovascular devices are for monitoring purposes and find their applications in the wellness and fitness sector. Cardiovascular devices have the potential to aid healthcare practitioners in diagnosing and even predicting cardiovascular disease. This can be accomplished using artificial intelligence (AI) and machine learning (ML) algorithms. The use of AI&ML can also help in improving existing CWDs. This section highlights some of the recent efforts made in the use of AI&ML for cardiovascular disease.

**Figure 6:**
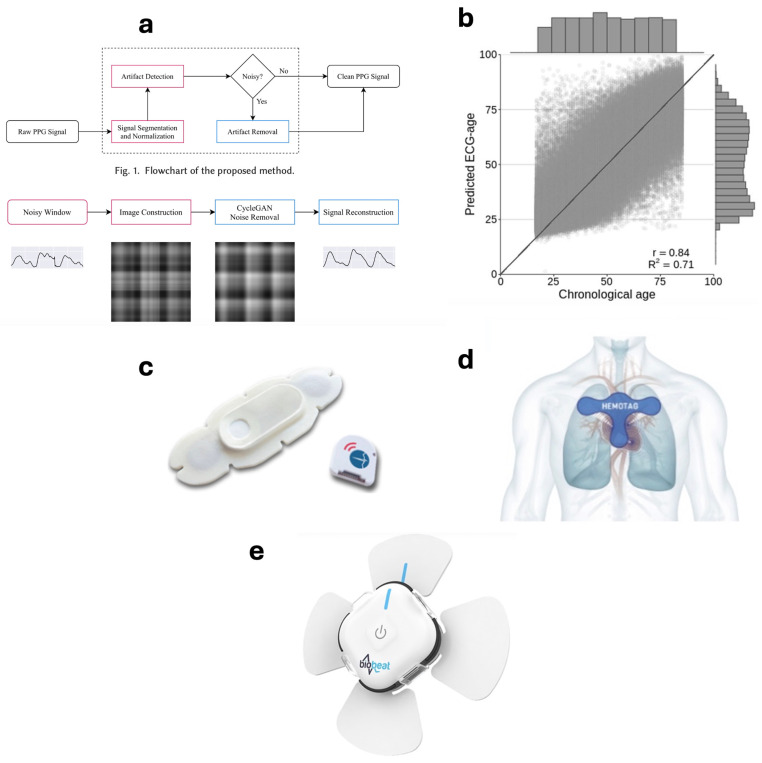
(**a**) Flowchart for the removal of motion artifact using Cycle Generative Adversarial Network (CycleGAN) [71]. (**b**) Results from the age predicted from the ECG using deep neural network [74]. (**c**) A wearable sensor from VitalConnect to measure multiple parameters for training a machine learning algorithm for heart failure event prediction [76]. (**d**) Hemotag for measuring aortic, pulmonic, and sternum waveforms [78]. (**e**) Biobeat skin patch for measuring multiparametric vitals [80].

It has been discussed how accelerometers are commonly used to remove motion artifacts from the cardiovascular parameters. However, AI&ML can be used to remove motion artifacts without accelerometers. For example, Zargari et al. have used machine learning to remove motion artifacts from the PPG signal for measuring BP, RR, and HRV [71]. They have reconstructed the clean PPG signal from the noisy PPG signal using Cycle Generative Adversarial Network (CycleGAN) [71]. CycleGAN is an unsupervised learning technique for translating the learning from one dataset (input dataset) into the target dataset (desired dataset). The flowchart of the overall algorithm is shown in Figure 6a. The technique was able to remove motion artifacts 9.5 times better than the commonly used accelerometer and was able to achieve a 45% efficiency in energy consumption [71]. Moreover, Lima et al. have used ML to predict a patient’s age using their ECG. For this purpose, they have used a deep neural network model that has been modeled using 12-lead ECG from 1,558,415 patients [72]. Figure 6b shows the results of the predicted age vs. the chronological age. The AI analysis leads to prognostic information highlighting the prediction of the mortality rate of patients [72]. According to the analysis, patients with the predicted age from the ECG more than 8 years greater than their chronological age have a higher mortality rate whereas patients with their predicted age more than 8 years smaller than their chronological age have a lower mortality rate [72].

Similarly, Zhu et al. have used ECG to train an ML model for the automatic labeling of ECG [73]. In this study, more than 180,000 12-lead ECGs of more than 70,000 patients were used to train a convolutional neural network (CNN). The model was trained to detect 21 unique heart rhythms. The automatically labeled ECGs were compared with the standard ECG labeled by cardiologists and the model was found to perfectly label 80% of test ECGs with 99.5% specificity, 86.7% sensitivity, and 98.3% mean area under the curve (AUC) of the receiver operating characteristic (ROC) score [74]. Furthermore, unlike a traditional 12-lead ECG, Hannun et al. have used a single-lead ECG to train a deep neural network in order to classify 12 heart rhythms [73]. The ECGs were obtained from a wearable device, the Zio^®^ monitor, by iRhythm (San Francisco, CA, USA) [75]. The model achieved a 0.97 ROC score. In another study by Stehlik et al., a predictive algorithm was developed by using a wearable sensor from VitalConnect (CA, USA), as shown in Figure 6c [76]. The algorithm is based on multiple parameters including HRV, HR, walking, gross activity, activity, body posture, and tilt [76]. The algorithm generated alerts to predict HF events around 6.5 days before the hospital readmission along with 85% specificity and 76–88% sensitivity for HF exacerbation precursors [76].

Using echocardiography, Asch et al. implemented an artificial intelligence model to predict mortality using left ventricular ejection fraction (LVEF) and left ventricular longitudinal strain (LVLS) [77]. For this purpose, LVEF and LVLS were obtained from the transthoracic echocardiography of patients with COVID-19 and an automated software based on AI named EchoGo (https://www.ultromics.com/press-releases/ultromics-launches-echogo-core-2.0, accessed on 21 July 2024) [77]. EchoGo is a cloud-based software that uses AI to contour the left ventricular (LV) echocardiogram and automatically performs Simpson’s calculation [77]. EchoGo is based on two AI models, an auto-contouring model and a view classifier. Both these models are based on two-dimensional convolutional neural networks [77]. The study found EchoGo to be an effective tool in performing AI-based analysis for predicting in-hospital and follow-up mortality. Similarly, Hemotag (FL, USA) is another echocardiogram-based sensor that has been found to be useful for measuring cardiac vitals [78]. Hemotag, as shown in Figure 6d, measures aortic, pulmonic, and sternum waveforms using a quad-sensor. This quad-sensor measures time-synchronized vibrations in order to measure cardiac time intervals (CTIs) [78]. In one study, Hemotag has been found to relate elevated CTIs with the identification of acutely decompensated heart failure (ADHF) [79].

Moreover, Biobeat (USA) is a PPG-based multi-parametric sensor that measures 13 different vitals [80]. These vitals include heart rate variability, pulse pressure, blood pressure, respiration rate, pulse rate, blood saturation, systemic vascular resistance, mean arterial pressure, stroke volume, cardiac output, cardiac index, and skin temperature [80]. It is available in two different designs in the form of a wristwatch and in the form of a skin patch where the skin patch also allows one-lead ECG. A Biobeat skin patch is shown in Figure 6e. Biobeat measures these vitals continuously and in real-time and produces an early warning score with a customized threshold to generate an alert whenever one of the parameters is below their set thresholds [80].

## 6. Future Perspectives

Cardiovascular devices have been instrumental in treating and monitoring the progression of cardiovascular disease. Their efficacy has increased manifold with the advancements in wearable devices. However, most of the current CWDs are used for monitoring purposes and their utility will increase if they can be used for diagnostic and prognostic purposes. As previously discussed, some efforts have been made recently to use AI&ML algorithms for this purpose but currently, most are used as post-processing techniques. It would be beneficial if CWDs could be programmed with AI&ML. This will not only speed up diagnostic results but will also conserve power consumption and other post-processing resources. An alert generation algorithm for heart failure is one good step in this direction. Moreover, there is a need to expand the applicability of the CWDs and one potential avenue would be to use CWDs to evaluate the change in the emotional state of the wearer using changes in the cardiovascular parameters. For example, heart rate, blood pressure, and respiratory rate often increase in the state of anxiety and hence can be used as diagnostic parameters for anxiety [81].

Furthermore, there is a need for energy-efficient CWDs to increase their duration of use. Longer device use duration means CWDs can be implemented on a larger scale by more patients. One possible solution is the development of self-powered CWDs. Self-powered CWDs can harvest power from the environment or from the body itself [82]. Self-powered CWDs will not only increase the duration of the data acquisition from the CWDs but will also help in miniaturizing them, which will increase the wearers’ compliance and comfortability. Some of the existing self-powered technologies include pyroelectric nanogenerators, biofuel cells, and piezoelectric nanogenerators [83].

Pyroelectric nanogenerators are nanomaterials that use the pyroelectric effect to convert thermal energy into electric energy where the pyroelectric effect is due to continuous changes in the polarization of crystals due to changes in their temperature [83]. On the other hand, the piezoelectric effect is the conversion of mechanical energy into electrical energy. Biofuel cells refer to cells that generate biochemical energy using oxidation and reduction reactions [83]. These techniques can be used to develop self-powered CWDs. For example, the piezoelectric effect can be used to convert heart contractions into electrical energy [83]. Similarly, photovoltaic effects can be used to harvest solar energy. For this purpose, efforts were made in the past to power pacemakers with solar cells [82]. However, the efficiency of implantable solar cells decreases with the increase in the depth of the implant; therefore, further research is required for this technique to be clinically practical [83].

Many advances have been made in telehealth monitoring in which Internet of Things (IoT) designs have been used to share biometric and physiological data over cloud databases. This type of monitoring or post-processing purpose is seen in the case of AI&ML algorithms [84]. One concern is that the sharing of private data increases the risk of data breaches and data spills. This can lead to potential damage to the wearers’ privacy and needs further attention and more secure communication protocols.

## 7. Conclusions

Cardiovascular devices for monitoring and treatment have evolved over the years. This paper reviews advances in cardiovascular devices starting from the development of implantable cardiovascular devices to the technological growth resulting in wearable devices. The paper also discusses the application of artificial intelligence algorithms for cardiovascular devices along with some future considerations. These considerations include the use of artificial intelligence for diagnosis and prognosis purposes in real-time instead of mere post-processing techniques and the use of self-powered cardiovascular devices along with more secure data-sharing protocols.
